# Acute elevated platform triggers stress induced hyperalgesia and alters glutamatergic transmission in the adult mice anterior cingulate cortex

**DOI:** 10.1016/j.ibneur.2020.12.002

**Published:** 2021-02-03

**Authors:** Koki Kawakami, Kohei Koga

**Affiliations:** a Department of Neurophysiology, Hyogo College of Medicine, Nishinomiya, Hyogo 663-8501, Japan; b Department of Biomedical Chemistry, School of Science and Technology, Kwansei Gakuin University, 2–1 Gakuen, Sanda, Hyogo 669-1337, Japan

**Keywords:** Stress induced hyperalgesia, Elevated open platform, Anterior cingulate cortex, Whole-cell patch-clamp recording

## Abstract

Pain is composed of both physiological and affective/emotional components which potentiate one another. In addition, exposure to stress modulates pain and affective behaviors including, anxiety-like behavior and/or depression-like behaviors. Indeed, chronic exposure to stress has been known to enhance stress-induced hyperalgesia (SIH). The anterior cingulate cortex (ACC) is critically involved in pain sensation and emotions. Animal models of chronic pain, but not acute nociception have been found to induce synaptic plasticity on glutamatergic and GABAergic transmission in the rodent ACC. However, it is unclear whether acute stress exposure could produce SIH and cause synaptic plasticity in the ACC. Accordingly, we studied how acute exposure of stress by the elevated open platform (EOP) could affect mechanical threshold, thermal and cold latency in the adult mice. Thirty minutes of the EOP produced mechanical hypersensitivity lasting for 60 min and thermal hypersensitivity immediately after the exposure. Next, we tested whether the stress could alter the excitatory and inhibitory synaptic transmission in the ACC. We performed whole-cell patch-clamp recordings from layer II/III pyramidal neurons in the ACC and analyzed both glutamatergic and GABAergic transmission in mice following the EOP. Thirty minutes of the EOP altered the rise and decay time of spontaneous glutamatergic AMPA/GluK receptors mediated currents, but did not change the frequency or amplitude of excitatory transmission. By contrast, the kinetics of inhibitory synaptic currents were not altered by the EOP. These results suggest that acute stress by the elevated platform produces SIH and causes synaptic plasticity on excitatory transmission, but not inhibitory transmission in the ACC.

## Highlights

• Mice exposed by 30 min after the elevated open platform showed mechanical hypersensitivity.
• The acute stress altered glutamatergic synaptic transmission, as indicated by changes in rise and decay time, in the ACC.
• GABAergic transmission was not altered by acute stress.

## Nomenclature

ACC: anterior cingulate cortex
EOP: elevated open platform
5-HT: serotonin: 5-hydroxytryptamine
NA: noradrenaline
sEPSCs: spontaneous excitatory postsynaptic currents
SIH: stress induced hyperalgesia
sIPSCs: spontaneous inhibitory postsynaptic currents
SSRI: selective serotonin reuptake inhibitors

## 1 Introduction

Pain as well as nociception share several comorbidities including physiological and affective/emotional aspects (Bushnell et al., 2013). Persistent nociceptive stimulation produces negative emotions such as anxiety and depression. In turn, these negative emotions facilitate pain sensation. Thus, physiological and emotional aspects of pain interact and potentiate one another (Bushnell et al., 2013). Human imaging and animal studies have consistently shown that the anterior cingulate cortex (ACC) is important for both the physiological and emotional aspects of pain (Bliss et al., 2016, Bushnell et al., 2013, Vogt, 2005). Nociceptive stimulation activates ACC neurons and animal models of chronic pain produce synaptic plasticity (Bliss et al., 2016, Vogt, 2005, Zhuo, 2016). *In vivo* whole-cell patch-clamp analysis shows that nociceptive stimulation evokes action potentials in layer II/III pyramidal neurons from the mouse ACC (Koga et al., 2010). Animal models of chronic pain, such as peripheral inflammation and nerve injury produce short-term and long-term synaptic plasticity on glutamatergic transmission (Zhuo, 2008, Zhuo, 2016). Additionally GABAergic transmission is also reduced in chronic pain models (Blom et al., 2014, Koga et al., 2018). Interestingly, a presynaptic form of long-term glutamatergic plasticity in the ACC is involved in anxiety like behaviors suggesting that there may be some overlap in functionality between pain and anxiety regions (Koga et al., 2015).

Stress is comorbid with the physiological sensation and emotional state of pain (Wall, 2013). Stress exposure triggers freezing behaviors and can induce hyperalgesia, known as stress-induced hyperalgesia (SIH) (Jennings et al., 2014). In addition, stress enhances affective states, such as anxiety- and depression-like behaviors (Steimer, 2002). Stress is classified as acute, developmental, and chronic based on the intensity and the duration of the exposure (de Kloet et al., 2005). Thus far, animal models with chronic exposure to stress have been used to understand the dramatic changes of physiological and emotional behaviors (Jennings et al., 2014). For example, the forced-swim test and/or restraint/immobilization stress induce SIH and emotional behaviors (Jennings et al., 2014). Importantly, prefrontal cortical areas including the ACC can be altered by stress exposure as revealed by anatomical and morphological studies. Indeed, stress exposure causes morphological changes of neurons in these cortical areas (Luczynski et al., 2015). The number of active neurons, as indicated by cFos is increased in the ACC (Moench et al., 2019). According to the morphological changes of neurons and the increased number of cFos positive neurons, it is possible that stress produces synaptic plasticity in the ACC. However, whether or not acute stress, which induces physiological and emotional responses, produces SIH and synaptic plasticity in the ACC has not been tested.

In this study, we used the elevated open platform (EOP) to test whether or not acute stress could produce SIH in mice. Furthermore, we performed whole-cell patch-clamp recordings from layer II/III pyramidal neurons in brain slice and analyzed if the stress could alter excitatory and inhibitory transmission in the ACC. These results provide direct evidence to connect acute stress, caused by SIH, produces synaptic plasticity in the ACC.

## 2 Experimental procedures

### 2.1 Animal

Male adult C57BL/6 mice (8–12 weeks old) were used in the experiment (SLC, Hamamatsu, Japan). Mice were housed at 23 ± 2 °C with a 12/12-h light/dark cycle (light on at 08:00 h) and were given free access to commercial food and tap water. All animal experimental procedures were approved by the Hyogo College of Medicine Committee on Animal Research (19−006) and were performed in accordance with the National Institutes of Health guidelines on animal care. Every effort was made to minimize animal suffering and reduce the number of animals used.

### 2.2 Behavioral tests (mechanical, thermal or cold stimulations)

**Fig. 1:**
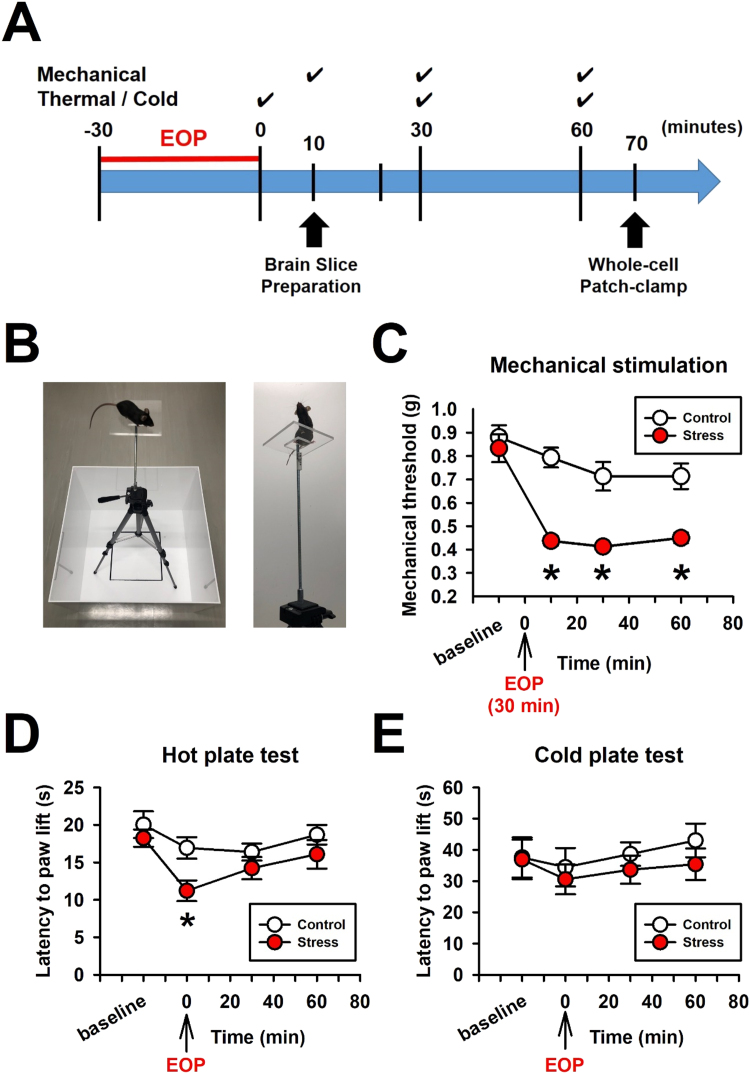
Acute stress produced mechanical hypersensitivity in mice. (A) Timeline of experiment; Mechanical thresholds were tested by von Frey filaments in mice as baseline. Mice were exposed by the elevated open platform (EOP) for 30 min. After 30 min of EOP, mechanical thresholds were measured in 10, 30 and 60 min intervals. Coronal brain slices were made after 30 min of EOP exposure for in vitro whole-cell patch-clamp recording analyses. (B). Photos of EOP: The elevated open platform apparatus was a square (10 cm × 10 cm) and transparent at a height of 1 m. Mice were exposed by the EOP for 30 min (C) Withdrawal thresholds (g) in response to mechanical stimulation in the left hindpaw as pre-stress exposure and after 10, 30, and 60 min of the EOP exposure (control: n = 20 mice, stress: n = 16 mice). (D)Latency of hindpaw withdrawal responses to hot-plate test at 52.5 °C. The thermal latency was reduced immediately after the EOP exposure (control; n = 8, 16.93 ± 1.43 s, stress; n = 8, 11.20 ± 1.38 s). (E) Latency to hindpaw withdrawal responses to cold-plate test at 4.0 °C (control; n = 8, stress; n = 8). Results were expressed as the mean ± SEM. *P < 0.05 compared stress to control at each time point (Two-way ANOVA). n.s. represents not significant (Two-way ANOVA, P > 0.05).

Mechanical threshold was measured before and after the elevated platform for 30 min, using the von Frey filament test (Fig. 1A) (Koga et al., 2015). Briefly, mice were placed in a plastic cage with a wire mesh bottom which permitted the experimenter full access to the paws. Behavioral accommodation was allowed for approximately 60 min until cage exploration. The intensity of the filament used was 0.16 g, 0.4 g, 0.6 g, 1.0 g, 1.4 g and 2.0 g to the left hind paw. Stimulation was applied in ascending order according to force (g). Threshold was set based on the minimum stimulation needed to evoke a withdrawal reflex (Koga et al., 2018).

Responses to noxious thermal stimulation or cold stimulation were assessed with a hot/cold plate test (thermal: 52.5 °C, cold: 4.0 °C, respectively, Ugo Basile, No. 35100). The onset of brisk hindpaw lifts and/or flicking/licking of the hindpaw were measured as a latency to withdrawalin each experiment. The cut-off time was determined at 60 s

### 2.3 Acute stress model induced by the elevated open platform (EOP)

Based on a previous study (Rocher et al., 2004), mice were exposed in the clear square Plexiglas board (10 cm × 10 cm) at a height of 1 m for 30 min (Fig. 1B). When the mice were placed on the EOP, they exhibit stress-voked responses such as freezing behaviors and/or anxiety-like behaviors. Before and after the 30 min of the EOP, we tested mechanical hypersensitivity in pre-stress exposure, 10, 30, and 60 min after the EOP. Control mice were returned to their home cages for 30 min and were tested by von Frey filaments. The mice that were exposed to 30 min of the EOP and demonstrated mechanical hypersensitivity were used for in vitro electrophysiological analysis.

### 2.4 In vitro whole-cell patch-clamp recording from the ACC neurons

Coronal brain slices (300 µm) at the level of the ACC were prepared as previously published (Koga et al., 2015, Koga et al., 2018). Briefly, brain slices were transferred to a submerged recovery chamber with oxygenated (95% O_2_ and 5% CO_2_) artificial cerebrospinal fluid containing (in mM) 124 NaCl, 2.5 KCl, 2 CaCl_2_, 1 MgSO_4_, 25 NaHCO_3_, 1 NaH_2_PO_4_, and 10 glucose at room temperature for at least 1 h. Experiments were performed in a recording chamber on the stage of an BX51WI microscope (Olympus, Tokyo, Japan) with infrared differential interference contrast optics for visualization. Recordings were performed from layer II/III pyramidal neurons in the ACC with a MultiClamp 700 A amplifier (Molecular Devices, Sunnyvale, CA) at room temperature. In the voltage-clamp configuration, recording electrodes (2–5 MΩ) contained the pipette solution composed of (in mM) 120 K-gluconate, 5 NaCl, 1 MgCl_2_ 0.5 EGTA, 2 Mg-ATP, 0.1 Na_3_GTP, and 10 HEPES; pH 7.2, 280–300 mOsmol were used for recording spontaneous excitatory post-synaptic currents (sEPSCs). The membrane potential was held at −70 mV for sEPSCs with a GABA_A_ receptors antagonist, picrotoxin (100 μM) in the bath solution. For recording spontaneous inhibitory postsynaptic currents (sIPSCs), 120 Cs-MeSO_3_ was added to the pipette solution instead of K-gluconate. To record sIPSCs, the membrane potential was held at 0 mV. Since picrotoxin abolishes the sIPSCs, the sIPSCs were GABA_A_ receptors mediated currents (Koga et al., 2018). To record membrane potential and action potentials, K-gluconate containing internal solution was used under the current-clamp mode. The EPSCs and IPSCs were measured by Mini Analysis software. Rise and decay time of sEPSCs and IPSCs were measured between 10% and 90% of amplitude. The initial access resistance was 15–30 MΩ and was monitored throughout the experiment. Data were discarded if the access resistance changed >15% during experiment. Data were filtered at 2 kHz and digitized at 10 kHz.

### 2.5 Statistics

Data are expressed as means ± SEM. Statistical analyses were done with Sigmaplot (12.5) software. Results were analyzed for significance with analysis of variance and post-hoc testing. The differences of behavioral and electrophysiological data among the groups were analyzed by one- way repeated-measures analysis of variance, followed by the Tukey-Kramer test for multiple comparisons or Student’s *t*-test for two comparisons. A value of P < 0.05 was taken to indicate a statistically significant difference.

## 3 Results

### 3.1 Acute stress by the EOP produced mechanical hypersensitivity

We first measured the mechanical threshold in mice with von Frey filaments before the EOP. The averaged thresholds between stress and control groups were similar before 30 min of the EOP (stress group: n = 16, 0.83 ± 0.06 g, control group: n = 12, 0.77 ± 0.06 g). After measuring the mechanical thresholds, the mice were placed on the EOP (Fig. 1B). After acute EOP exposure, we tested the mechanical threshold 10, 30 and 60 min. Interestingly, the acute stress exposure to EOP enhanced SIH for at least 60 min, while mice without EOP exposure were no different from the control group (two-way ANOVA, P < 0.05, the EOP versus control group, Fig. 1C). Indeed, 10 min after the EOP exposure, there was a peak in the SIH (stress: n = 16, 0.44 ± 0.02 g; control: n = 22, 0.74 ± 0.05 g, P < 0.05) (Fig. 1C). Furthermore, we tested if the 30 min EOP exposure could alter thermal and cold sensitivity (Fig. 1D). The hot plate test at 52.5 °C revealed that the EOP group responded with altered thermal latency (two-way ANOVA, P < 0.05, Fig. 1D). Moreover, immediately after exposure to EOP mice demonstrated thermal hypersensitivity (stress: n = 8, 11.20 ± 1.38 s; control: n = 8, 16.92 ± 1.43 s, P < 0.05) (Fig. 1D). In addition, we examined cold sensitivity in response to a cold plate test following 30 min of EOP exposure. The cold sensitivities were not different between the EOP and control group (stress: n = 8; control: n = 8, two-way ANOVA, P > 0.05, Fig. 1E). These results suggest that acute stress exposure of 30 min on an elevated platform produce hypersensitivity depending on the mode of afferent stimulation; with mechanical hypersensitivity lasting for at least 60 min and thermal hypersensitivity occurring immediately after EOP exposure. By contrast, cold sensitivity, was unaltered.

### 3.2 Acute stress by the EOP altered kinetics of glutamatergic transmission in ACC neurons

**Fig. 2:**
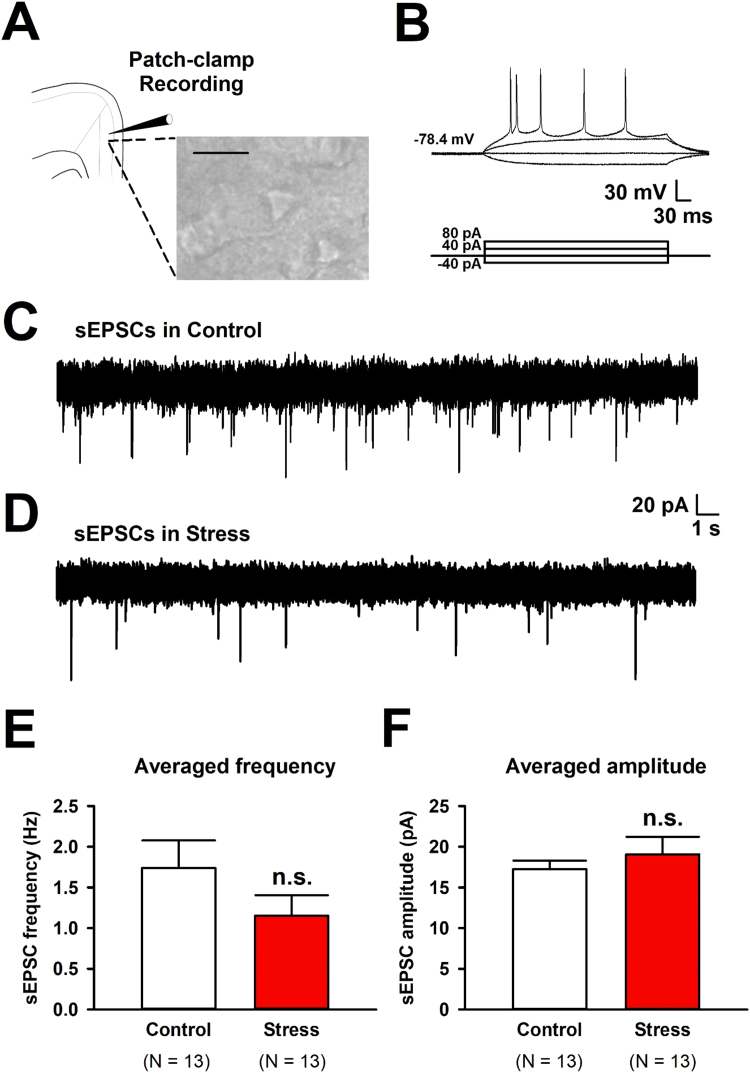
Frequency and amplitude of sEPSCs in pyramidal neurons from the ACC was not affected 30 min of EOP exposure. (A) The recording scheme of whole-cell patch-clamp recording in the ACC and a representative image of neurons from layer II/III in the ACC. Scale bar is 20 µm. (B)A typical trace of action potential firing pattern in a pyramidal neuron by current injection (−40, 0, 40, 80 pA for 400 ms). (C) A typical sEPSCs holding membrane potential at −70 mV from the control group. (D)Grouped data for recorded sEPSCs after 30 min of EOP exposure. (E) Averaged frequency of sEPSCs in control (n = 13 neurons/12 mice, 1.74 ± 0.34 Hz) and stress groups (n = 13/11, 1.15 ± 0.25 Hz). (F) Averaged amplitude of sEPSCs in control (n = 13/12, 17.26 ± 1.03 pA) and stress groups (n = 13/11, 19.06 ± 2.16 pA). Results were expressed as the mean ± SEM. n.s. represents not significant (unpaired *t*-test, P > 0.05).

**Fig. 3:**
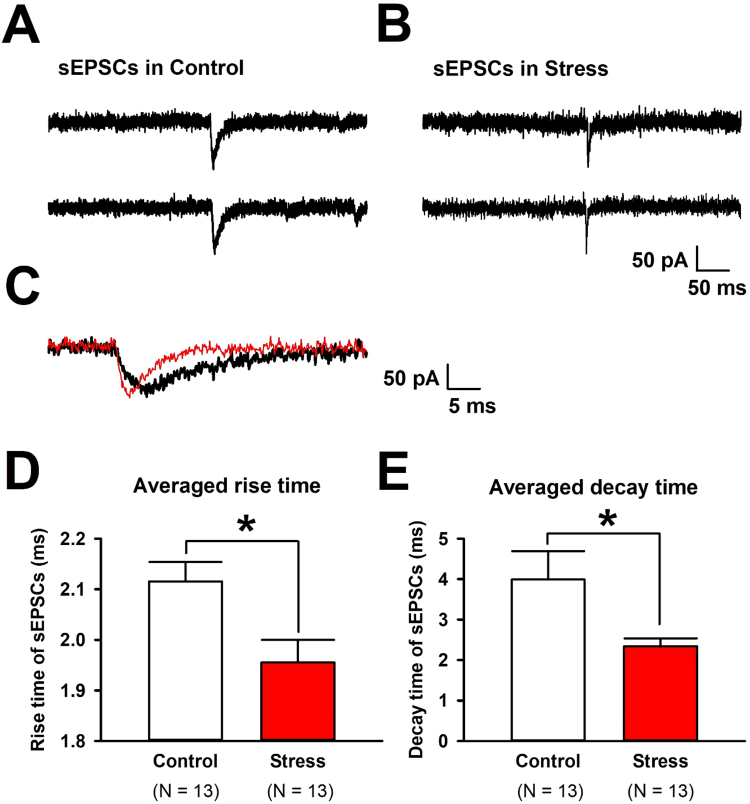
Both rise and decay time of sEPSCs in the layer II/III pyramidal neurons from the ACC were altered after EOP exposure. (A) The expanded traces of sEPSCs in control group. (B)Representative high-magnification of sEPSCs in stress group. (C) The superimposed traces of sEPSCs in control (black) and stress (red) groups. (D)Averaged rise time of sEPSCs in control (n = 13 /12, 2.12 ± 0.04 ms) and stress (n = 13/11, 1.96 ± 0.04 ms) groups. Stress group showed shorter rise time of sEPSCs compared with control group. Averaged decay time of sEPSCs in control (n = 13/12, 3.99 ± 0.70 ms) and stress (n = 13/11, 2.34 ± 0.19 ms) groups. Results are expressed as the mean ± SEM. *P < 0.05 compared stress to control (unpaired *t*-test, P > 0.05).(For interpretation of the references to color in this figure legend, the reader is referred to the web version of this article.)

Since it took at least 10 min of EOP exposure to produce a peak in SIH, we tested if the ACC neurons altered glutamatergic transmission under acute stress condition in mice (Fig. 2). We made coronal brain slices immediately after the EOP exposure and then, we performed whole-cell patch-clamp recordings from layer II/III pyramidal neurons in the adult mouse ACC to examine synaptic transmission. Firing patterns of pyramidal neurons could be recorded since we used K-gluconate based internal solution (Fig. 2A, B). Since sEPSCs were completely blocked by α-amino-3-hydroxy-5-methyl-4-isoxazolyle propionic acid (AMPA)/GluK receptors antagonist, 6-cyano-7-nitroquinoxaline-2,3-dione (CNQX, 10 μM), the EPSCs were AMPA/GluK receptors mediated currents (Wu et al., 2005, Zhao et al., 2006). We analyzed AMPA/GluK receptors mediated spontaneous excitatory postsynaptic currents (sEPSCs) in control and stress groups (control: n = 13 neurons/12 mice; stress: n = 13 neurons/11 mice, Fig. 2C, D). The average frequency of sEPSCs was not changed between stress and control group (stress: 1.15 ± 0.25 Hz; control: 1.74 ± 0.34 Hz, unpaired *t*-test, P > 0.05, Fig. 2E). The average amplitude of sEPSCs was not altered between these groups (stress: 19.06 ± 2.16 pA; control: 17.26 ± 1.03 pA, unpaired *t*-test, P > 0.05, Fig. 2F). We further analyzed the kinetics of AMPA/GluK receptors mediated currents (Fig. 3). Interestingly, the average rise time and decay time of sEPSCs were changed in stress group (rise time in stress: 1.96 ± 0.04 ms; in control: 2.12 ± 0.04 ms, unpaired *t*-test, P < 0.05, Fig. 3A-D, decay time in stress: 2.34 ± 0.19 ms; in control: 3.99 ± 0.70 ms, unpaired *t*-test, P < 0.05, Fig. 3A-C, E). These results suggest that acute exposure of the EOP alter the kinetics of AMPA/GluK receptors in the ACC neurons.

### 3.3 Inhibitory transmission is insensitive to the acute stress

**Fig. 4:**
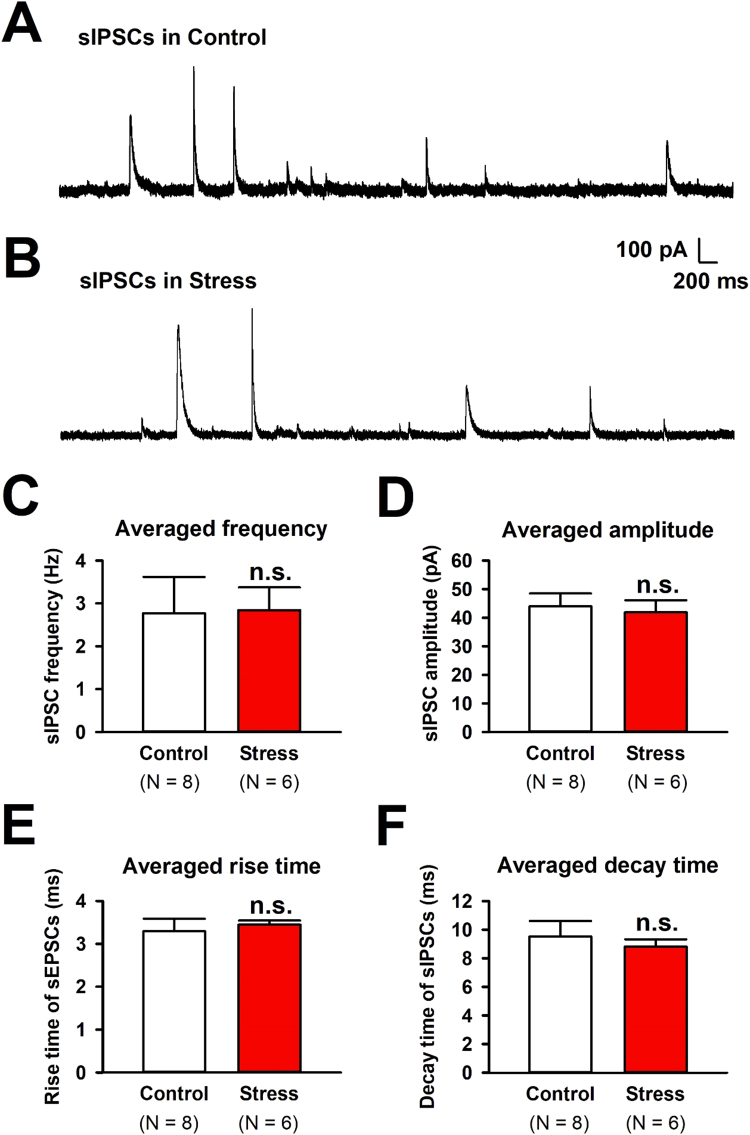
Inhibitory transmission in the ACC was not affected by 30 min of EOP exposure. (A) A typical trace of sIPSCs holding membrane potential at 0 mV in a layer II/III pyramidal neuron in a control group. (B) Representative sIPSCs, recorded after 30 min of EOP exposure, in the stress group. (C) Averaged frequency of sIPSCs in control (n = 8 /8, 2.77 ± 0.84 ms) and stress (n = 6/6, 2.84 ± 0.53 ms) groups. (D) Averaged amplitude of sIPSCs in control (n = 8/8, 43.99 ± 4.45 pA) and stress (n = 6/6, 41.85 ± 4.25 pA) groups. (E) Averaged rise time of sIPSCs in control (n = 8/8, 3.29 ± 0.30 ms) and stress (n = 6/6, 3.45 ± 0.10 ms) groups. (F) Averaged decay time of sIPSCs in control (n = 8/8, 9.52 ± 1.09 ms) and stress (n = 6/6, 8.82 ± 0.51 ms) groups. Results are expressed as the mean ± SEM. n.s. represents not significant (unpaired *t*-test, P > 0.05).

Next, we analyzed if acute stress by EOP exposure could alter inhibitory transmission in the ACC (Fig. 4). We analyzed GABA_A_ receptors mediated sIPSCs in control and stress groups (control: n = 8/8, stress: n = 6/6, Fig. 4A, B). The stress exposure did not change the average frequency of sIPSCs (stress: 2.84 ± 0.53 Hz; control: 2.77 ± 0.84 Hz, unpaired *t*-test, P > 0.05, Fig. 4C). The average amplitude of sIPSCs were not different between control and stress groups (stress: 41.85 ± 4.25 pA; control: 43.99 ± 4.45 pA, unpaired *t*-test, P > 0.05, Fig. 4D). The average kinetics of rise time and decay time in sIPSCs were not altered between control and stress groups (rise time in stress: 3.45 ± 0.10 ms; rise time in control: 3.29 ± 0.30 ms, decay time in stress: 8.82 ± 0.51 ms; decay time in control: 9.52 ± 1.09 ms, unpaired *t*-test, P > 0.05, Fig. 4E and F). These results indicate that acute stress is insensitive to GABA_A_ receptor-mediated inhibitory transmission in the ACC.

## 4 Discussion

In this study, we examined whether acute stress exposure induced by EOP could cause mechanical hypersensitivity and alter synaptic transmission in the adult mouse ACC, known to play a crucial role in pain and emotion. We found that mice exposed as long as 30 min to the EOP showed mechanical hypersensitivity which peaked at 10 min and lasted for at least 60 min. Furthermore, the kinetics of glutamatergic AMPA/GluK receptors mediated transmission were altered without affecting frequency or amplitude of sEPSCs in the layer II/III pyramidal neurons from the ACC. In contrast, GABAergic transmission was unchanged by the acute exposure in the EOP.

### 4.1 Elevated platform produced stress induced mechanical and thermal hypersensitivity and cold

Stress is known to have an influence on nociception. In some reports, acute stress can induce analgesia (Butler and Finn, 2009). By contrast, chronic stress can induce hyperalgesia (Jennings et al., 2014). For instance, major chronic stress models such as forced swim test and restraint/immobilization stress produce SIH (Jennings et al., 2014). It was still unclear whether or not acute stress, by EOP exposure, would cause SIH. In this study, we found that acute EOP exposure for 30 min produced SIH. The short time exposure of stress caused mechanical hypersensitivity for at least 60 min. Importantly, the elevated platform apparatus, including the EOP, produce freezing behaviors (Miyata et al., 2011, Miyata et al., 2007). The EOP also increases the release of stress related substances including corticosterone and serotonin (5-HT) (Miyata et al., 2011, Rocher et al., 2004). Since, pain and/or nociceptive sensation are composed of physiology and emotional aspects, and these two aspects potentiate each other, the enhanced stress responses of freezing behaviors and/or releasing stress hormone by the EOP may trigger SIH (Bushnell et al., 2013, Jennings et al., 2014, Lupien et al., 2009).

The hippocampus, amygdala and prefrontal cortex are all involved in stress related behaviors (Knudsen et al., 2018). In addition to synaptic plasticity in the ACC, stress exposure produces synaptic plasticity in hippocampus and amygdala (Lupien et al., 2009). Anatomically, neurons in hippocampus and amygdala project to prefrontal cortex and the ACC reciprocally (Lupien et al., 2009). Thus, it is possible that stress exposure could alter the brain area related to pain as well as emotion. The axons within the ACC project to the spinal cord directly and/or indirectly, are involved in descending facilitation (Chen et al., 2018). Importantly, stress exposure by repeated forced-swim test increases the release of glutamate and reduces GABA in spinal cord (Quintero et al., 2011). Thus, in part, the established synaptic plasticity on the glutamatergic transmission in the ACC might contribute to SIH by modulating spinal processing (Chen et al., 2018).

### 4.2 Elevated platform-induced stress alters synaptic plasticity in brain

Human imaging and animal studies have shown that the hippocampus, amygdala, and ACC are major regions that are altered by stress (Knudsen et al., 2018). Chronic stress often induces synaptic plasticity as evidenced by altered glutamatergic and GABAergic transmission in these stress-related brain areas. In chronic stress models, such as forced swim test and chronic restraint/immobilization stress, the synaptic plasticity can occur as a consequence of changes to both glutamatergic and GABAergic transmission (Bains et al., 2015, Krugers et al., 2010, Popoli et al., 2011). For the stress-induced synaptic plasticity in the ACC, chronic restraint stress produces synaptic plasticity on GABAergic transmission (Ito et al., 2010). Here, we found that acute stress, induced by the EOP, produced synaptic plasticity mainly on glutamatergic AMPA/GluK receptors mediated transmission, but not on GABAergic transmission in the ACC. In this study, we did not focus on NMDA receptors, which are also major glutamatergic receptors (Bliss et al., 2016). It is possible that EOP could alter NMDA receptor-mediated functions, and could be the focus of future study. In the acute stress model, the balance of synaptic excitation and inhibition is altered in the ACC. In support of our results, exposure to stress is able to produce morphological changes of neurons in the prefrontal area (Luczynski et al., 2015). This exposure also increases an activity-dependent gene, *cfos* in the prefrontal cortex (Moench et al., 2019). Since the ACC is related to pain and emotion and is a primary target of stress, the synaptic plasticity on glutamatergic transmission in postsynaptic AMPA/GluK receptors may contribute to produce SIH.

### 4.3 The mechanisms of glutamatergic plasticity by the SIH in the ACC

How the EOP alters the kinetics of glutamatergic AMPA/GluK receptors in the ACC is still unclear. However, it might be possible that stress hormones or transmitters change the kinetics of AMPA/GluK receptors. For example, glucocorticoid, 5-HT and noradrenaline (NA) are well-known to be involved in stress (Bains et al., 2015, Inoue et al., 2013, Miyata et al., 2011, Petty et al., 1997). Our preliminary data (not published here) indicates that systemic antagonism of glucocorticoid and mineralocorticoid receptors cannot abolish SIH in response to EOP exposure. Thus, 5-HT, NA and/or other stress related hormone may be other possible targets to enhance mechanical hypersensitivity and alter glutamatergic transmission in the ACC. In fact, 5-HT modulates glutamatergic transmission in layer II/III pyramidal neurons in the ACC (Tian et al., 2017). Furthermore, NA fibers from the locus coeruleus also project to the ACC (Koga et al., 2020). Recently, we reported that activation of NA in the ACC produced synaptic plasticity on glutamatergic transmission in the layer II/III pyramidal neurons (Koga et al., 2020). Therefore, the EOP could activate serotonergic/noradrenergic neurons and increases release of 5-HT and/or NA in the ACC. Accordingly, releasing 5-HT or NA induced by the EOP might sensitize AMPA/GluK receptors through phosphorylation of receptors in the ACC. Further study is needed to understand how acute stress produces glutamatergic plasticity in the ACC.

## CRediT authorship contribution statement

Kawakami K performed behavioral tests and electrophysiological analysis. Koga K designed the experiments and wrote the manuscript.

## Ethical statement

We comply with all ethical standards regarding animal experiments as indicated in the Methods section.

## Conflicts of Interest

We also declare no conflict of interests.
